# The Effectiveness of Prompts to Promote Engagement With Digital Interventions: A Systematic Review

**DOI:** 10.2196/jmir.4790

**Published:** 2016-01-08

**Authors:** Ghadah Alkhaldi, Fiona L Hamilton, Rosa Lau, Rosie Webster, Susan Michie, Elizabeth Murray

**Affiliations:** ^1^ eHealth Unit Research Department of Primary Care and Population Health University College London London United Kingdom; ^2^ Research Department of Clinical, Educational and Health Psychology University College London London United Kingdom

**Keywords:** systematic review, adherence, engagement, prompts, digital interventions

## Abstract

**Background:**

Digital interventions have been effective in improving numerous health outcomes and health behaviors; furthermore, they are increasingly being used in different health care areas, including self-management of long-term conditions, mental health, and health promotion. The full potential of digital interventions is hindered by a lack of user engagement. There is an urgent need to develop effective strategies that can promote users’ engagement with digital interventions. One potential method is the use of technology-based reminders or prompts.

**Objective:**

To evaluate the effectiveness of technology-based strategies for promoting engagement with digital interventions.

**Methods:**

Cochrane Collaboration guidelines on systematic review methodology were followed. The search strategy was executed across 7 electronic databases: the Cochrane Central Register of Controlled Trials (CENTRAL), MEDLINE, Embase, Web of Science, the Education Resources Information Center (ERIC), PsycINFO, and the Cumulative Index to Nursing and Allied Health Literature (CINAHL). Databases were searched from inception to September 13, 2013, with no language or publication type restrictions, using three concepts: randomized controlled trials, digital interventions, and engagement. Gray literature and reference lists of included studies were also searched. Titles and abstracts were independently screened by 2 authors, then the full texts of potentially eligible papers were obtained and double-screened. Data from eligible papers were extracted by one author and checked for accuracy by another author. Bias was assessed using the Cochrane risk of bias assessment tool. Narrative synthesis was performed on all included studies and, where appropriate, data were pooled using meta-analysis. All findings were reported according to the Preferred Reporting Items for Systematic Reviews and Meta-Analyses (PRISMA) guidelines.

**Results:**

A total of 14 studies were included in the review with 8774 participants. Of the 14 studies, 9 had sufficient data to be included in the meta-analyses. The meta-analyses suggested that technology-based strategies can potentially promote engagement compared to no strategy for dichotomous outcomes (relative risk [RR] 1.27, 95% CI 1.01-1.60, I^2^=71%), but due to considerable heterogeneity and the small sample sizes in most studies, this result should be treated with caution. No studies reported adverse or economic outcomes. Only one study with a small sample size compared different characteristics; the study found that strategies promoting new digital intervention content and those sent to users shortly after they started using the digital intervention were more likely to engage users.

**Conclusions:**

Overall, studies reported borderline positive effects of technology-based strategies on engagement compared to no strategy. However, the results have to be interpreted with caution. More research is needed to replicate findings and understand which characteristics of the strategies are effective in promoting engagement and how cost-effective they are.

## Introduction

Digital interventions (DIs) are programs that provide information and support—emotional, decisional, and/or behavioral—for physical and/or mental health problems via a digital platform (eg, website or computer) [1]. There has been substantial investment in DIs in developed countries, and they have been used in different health domains, including self-management of long-term conditions [2-4], promotion of healthy behaviors [1,5-7], and mental health [8]. The literature suggests that they can improve health behaviors and health outcomes [1-10]; however, systematic reviews of the effectiveness of DIs tend to report small effect sizes with a substantial level of heterogeneity [2,4,6,7,9]. One potential cause for the relatively small effect sizes is nonuse, or insufficient use, of the digital interventions [7]. Research has shown that there is a lack of engagement with DIs, and some studies have suggested a dose-response relationship between DIs’ effectiveness and a user’s level of engagement [11-16]. A review of DIs targeting physical activity showed that better engagement was associated with larger effects of the intervention [14]. Similar findings were seen in studies of DIs targeting fruit and vegetable consumption [11], weight loss [12], and smoking cessation [13,15]. Although it could be argued that the association between greater engagement and bigger positive effect is due to reverse causality (ie, the user experiences better outcomes so becomes more engaged), it is also plausible that better engagement leads to greater effectiveness [11-16]. Indeed, one systematic review of reviews looking at DIs aimed at health prevention reported, “One of the most substantial problems in online prevention is the low use of the interventions, a phenomenon seen across all behavior domains” [7]. Hence, one potential way of improving their effectiveness may be by promoting users’ engagement.

In a three-round systematic Delphi experiment done by Brouwer et al [17], engagement was conceptualized into three phases. In the first phase, the user decides to first visit a DI to determine what it offers and whether he/she can benefit from it. In the second phase—prolonging the first visit—a user extends this visit and is exposed to part of the DI. In the third phase—revisiting the DI—the user returns to the DI after the first visit. The Brouwer et al study suggested that different factors impact on each phase. During the first phase, factors influencing the decision whether or not to visit the DI for the first time include user characteristics (eg, motivation and interest) and perceived relevance of the DI. In the second phase, the duration of the first visit is mostly determined by the characteristics of the DI (ie, whether it is tailored and easy to use). In the third phase, the decision whether to revisit is influenced by both user characteristics, such as motivation, and the presence or absence of reminders or prompts to revisit [17]. This systematic review targets the third phase by exploring the use of prompts as a method to promote revisiting DIs after the first visit [7,18-20]. Some systematic reviews have been published about technology-based prompts; however, these reviews have focused on the effect of prompts on the behavior addressed by the DI, rather than on the proximal effect on engagement [21-23]. There is some emerging evidence on design features, including use of prompts, that influence engagement [19,24]; one systematic review that performed qualitative analysis of the results of the included studies found that DIs that used email and phone contact with users were more likely to have better engagement [25].

To our knowledge, none of those reviews has focused specifically on the relationship between engagement, prompts, and the characteristics of prompts. Characteristics likely to influence effectiveness include timing (ie, when should a prompt be used), duration (ie, for how long should it be used) [18,25-27], frequency [22], mode of delivery (eg, email, text message, or telephone call [23]), sender [28,29], content [30], and theoretical underpinning [23]. It has been shown that an intervention based on theory is more effective than one that is not [23,31].

A review of digital interventions found that those that used more behavior change techniques (BCTs) were more effective than those that used fewer BCTs [23]. Therefore, this review attempted to code the content of the prompts using a BCT taxonomy [32], the same one used by the previously mentioned review [23]. The BCT taxonomy, comprised of 93 BCTs, has been rated, grouped, and agreed on by international behavior experts in a Delphi-type study; these BCTs are defined as “observable, replicable, and irreducible components of an intervention designed to alter or redirect causal processes that regulate behavior” [32]. This taxonomy can help identify the active ingredients that the intervention contains and, thus, the mechanism of action, which allows for a theory-based explanation of how to develop prompts that are effective in promoting engagement. The BCT taxonomy includes the prompt/cue techniques that “introduce or define environmental or social stimulus with the purpose of prompting or cueing the behavior.” Thus, the term *strategy* was used in this review as it is more comprehensive and adaptable, and a strategy’s content can include the BCT prompt/cue or more components.

The aim of this systematic review was to evaluate the effectiveness of technology-based strategies, defined in this review as digital and analog technology methods used to promote the user’s regular interaction with all or part of the DI. These include, but are not limited to, emails, text messages, multimedia messages, telephone calls, automated voice calls, or faxes. Specific objectives of the review were to (1) describe technology-based strategies to promote engagement with DIs, (2) assess the effectiveness of technology-based strategies in promoting engagement with DIs, (3) explore whether different characteristics such as timing, duration, frequency, mode of delivery, sender, content, or use of theory are associated with differential effectiveness, and (4) to describe the cost of technology-based strategies to promote engagement with DIs.

## Methods

This review followed Cochrane methodological guidance for systematic reviews [33] and the protocol with the full details about the methodology has been published [34].

### Data Sources and Search Methods

The search was performed in 7 electronic databases: the Cochrane Central Register of Controlled Trials (CENTRAL), MEDLINE, Embase, Web of Science, the Education Resources Information Center (ERIC), PsycINFO (including studies and dissertation abstracts), and the Cumulative Index to Nursing and Allied Health Literature (CINAHL). Databases were searched from inception to September 13, 2013, with no language or publication type restrictions, using three concepts: randomized controlled trials (RCTs) *and* digital interventions *and* engagement (see Multimedia Appendix 1 for the MEDLINE search strategy). The search also included screening grey literature (Conference Proceedings Citation Index, formerly ISI Proceedings), references of the included studies, issues of key journals such as the Journal of Medical Internet Research (JMIR), and using Google Scholar to screen any papers citing included or other key papers [18,20,22,23].

### Article Screening and Selection

All citations identified by the search strategy were deduplicated and downloaded into Endnote X5 (Thomson Reuters). Titles and abstracts were screened by one author (GA) and were double-screened by one of 3 other coauthors (EM, FH, or RW). Full texts of potentially eligible articles were screened by 2 authors (EM and GA). Any disagreement was resolved through discussion, referencing the eligibility criteria. If consensus could not be achieved, a third author (FH) was consulted. Justifications for exclusion were recorded and tabulated. All reviewers had training in systematic review methodology.

### Inclusion Criteria

#### Participants

Participants were adults aged 18 years old or over. There were no limitations on gender, socioeconomic status, ethnicity, or health status. All settings were included for digital intervention; for technology-based strategies, the setting was online.

#### Interventions

The interventions of interest were technology-based strategies to promote engagement with digital interventions. To be included, the interventions had to meet the following definitions:

1. Digital interventions were defined as programs that provide information and support—emotional, decisional, and/or behavioral—for physical and/or mental health problems via a digital platform (eg, a website or a computer) [1].

2. Technology-based engagement-promoting strategies were defined as digital and analog technology methods used to promote the user’s regular interaction with all or part of the DI, including, but not limited to, telephones calls, text messages, multimedia messages, emails, automated voice calls, or faxes. Examples of interventions that were included were a computerized treatment program with mobile phone text messages that reminded the user to visit the program, and a blood pressure self-monitoring website that sent email prompts to users to enter their pressure readings on the website.

#### Comparisons

Three groups of comparators were defined: (1) minimal or inactive comparators, such as no strategy, (2) nontechnological strategies, such as printed materials or face-to-face contact, and (3) alternative technology-based strategies, for example, where the effects of email prompts are compared to the effects of text message prompts. Some studies tested the cumulative effect of multiple strategies; for example, both arms received prompts by email with one arm also receiving additional prompts by telephone call.

#### Outcomes

##### Primary Outcomes

The primary outcome was engagement with the DI, which was recorded as the number of log-ins/visits, number of pages visited, number of sessions completed, time spent on the DI, and number of DI components/features used. These measures were determined in advance before screening included studies [34].

##### Secondary Outcomes

Two types of secondary outcomes were selected:

1. Adverse outcomes, such as users feeling frustrated or irritated by email prompts, or experiencing a loss of self-esteem due to not being able to engage with the DI.

2. Economic outcomes, which were costs associated with strategies promoting engagement to inform future cost-effectiveness analysis.

#### Study Designs

RCTs were included; these were either trials of DIs that used strategies promoting engagement or trials evaluating strategies specifically. Economic evaluations were to be included if they were conducted alongside the main trial.

### Exclusion Criteria

The following were the exclusion criteria:

1. Interventions targeted exclusively at health professionals (eg, computer-based decision aids to assist health professionals in making decisions with regard to treatments).

2. Trials where attrition from the trial and disengagement from the DI are nondistinguishable.

3. Trials where the effect of the DI components cannot be separated from the effect of the engagement-promoting strategy (eg, trials where the DI is not compared to another DI, such as a website to lose weight with email prompts compared with dietician face-to-face sessions with emails from the dietician; or when the difference between the 2 arms included different DIs as well as differential engagement strategies).

In the protocol, it was stated that quasi-RCTs would be included; however, upon further reflection, and due to the reasonable number of eligible RCTs and the high risk of bias associated with quasi-RCTs, they were excluded.

### Data Extraction

Data were extracted from included papers using an adapted version of the Cochrane Consumers and Communication Review Group data extraction template. One author (GA) extracted all the included papers and another coauthor (FH) verified the accuracy of the extraction; any disagreement was resolved through discussion. If no agreement was reached, a third author (EM) was consulted. Authors were contacted for more information about the characteristics of the strategy and any missing outcome data. The taxonomy for the BCTs [32] was used; strategy contents were coded by one author (GA) during data extraction and verified by another author (RW), who is an experienced user of the taxonomy.

### Critical Appraisal Techniques

An assessment of risk of bias was done based on the Cochrane risk of bias assessment tool [33]. The following criteria were used:

1. Was the allocation sequence adequately generated?

2. Was allocation adequately concealed?

3. Was knowledge of the allocated interventions adequately prevented during the study (ie, blinding)?

4. Were incomplete outcome data adequately addressed?

5. Were study reports free of suggestion of selective outcome reporting?

6. Was the study free of other problems that could put it at risk of bias? These problems included, but were not limited to, baseline characteristic differences between groups, validity and reliability of outcome measures, sample size, and power.

The papers [11,35-47] were categorized as having low, high, or unclear risk of bias (ie, when the study did not provide enough information to judge the different aspects of trial quality). A risk of bias summary (see Multimedia Appendix 2A) and a risk of bias graph (see Multimedia Appendix 2B) were generated. The bias assessment was done by one author (GA) and was checked by another author (FH). Any discrepancies were resolved by a third author (EM).

### Data Synthesis

#### Selection of Outcomes

Outcome measures were categorized as dichotomous or continuous engagement outcomes:

1. Dichotomous engagement outcome: any dichotomous measure of how participants engaged with the DI, such as proportion of participants who visited the DI, or proportion of participants who completed a prespecified number of modules.

2. Continuous engagement outcome: any continuous measure of how participants engaged with the DI, such as number of visits or page views.

Even within the categories of dichotomous and continuous outcomes, authors often reported more than one outcome. After discussion with coauthors and for the purpose of analysis, one outcome was selected based on the following prespecified criteria:

1. The number of participants who visited the DI (ie, logged in to the website) or the number of visits/log-ins was selected, as these are the most appropriate indicators for engagement strategies [25,48].

2. The primary outcome defined or stated by the author.

3. The outcome reported separately for the control and intervention group, rather than lumped together.

4. The highest standard for engagement (ie, the authors report the number of participants who completed all the sessions rather than the number of participants who completed no sessions or a specific number of sessions).

5. Data from the longest measured follow-up period were chosen, as it is important to demonstrate sustained change.

#### Data Analysis

Results were reported according to the Preferred Reporting Items for Systematic Reviews and Meta-Analyses (PRISMA) guidelines [49] and analyzed according to Cochrane guidelines [33]. Data from included studies were tabulated to allow for a narrative description of the results. Data on characteristics of engagement strategies were tabulated and all authors of included studies were contacted for clarification about their strategies, of whom 4 replied [35-38].

A meta-analysis was performed and continuous and dichotomous data from RCTs were pooled separately using a random effects model. The appropriate effect measures were determined depending on the type of data. For dichotomous outcomes, relative risks (RRs) and their 95% confidence intervals were used. For continuous outcomes, standardized mean differences (SMDs) with 95% confidence intervals were used. Due to the variable nature of the interventions, heterogeneity was expected and it was assessed using the I^2^ statistic.

A sensitivity analysis was intended to be undertaken, as recommended by the Cochrane handbook, by excluding trials of poor quality to determine their effects on the study results, as well as a funnel plot to assess publication bias. However, there were insufficient studies to allow for a meaningful assessment. To investigate heterogeneity, a post hoc sensitivity analysis was conducted by removing one study [46] on the basis of visual inspection of the forest plots (see Multimedia Appendix 3).

## Results

### Summary of Search Results

**Figure 1:**
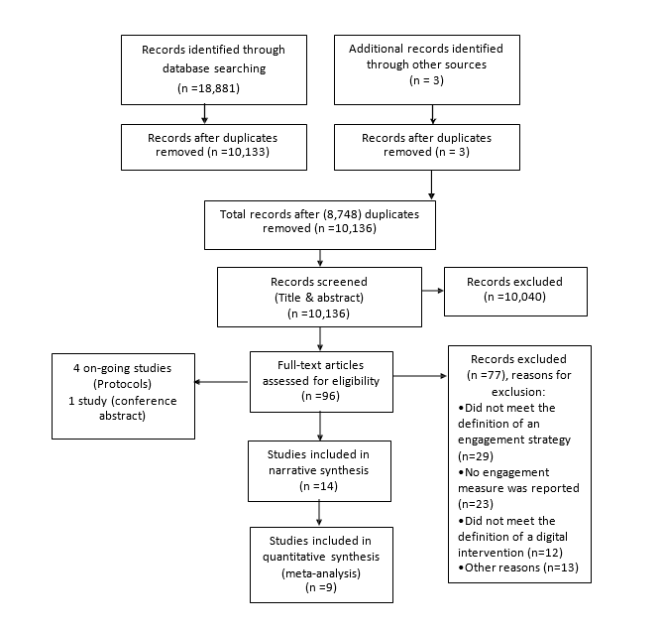
PRISMA flow diagram.

Searching the electronic databases yielded a total of 18,881 records. After removing all duplicates (manually and using Endnote X5), 10,133 records remained for title and abstract screening. Of these, 93 went forward for full-text assessment, supplemented by 3 studies identified from reference tracking. A total of 77 papers were excluded at full-text screening for various reasons, the most common being that the engagement strategy or DI did not meet the definition in this review, or that engagement was not measured in the study. There were 4 ongoing studies with only protocols available, and one study was a conference abstract. Figure 1 shows the results of the initial searches, screening, and selection processes.

### Included Studies

A total of 14 studies with 8774 participants were included in the systematic review; their characteristics are described in Table 1, with full details shown in Multimedia Appendix 4. The sample sizes ranged from 43 to 3448. One study was published in 2005 and the rest were published between 2009 and 2013; all studies were published in English. More than half of the studies [11,35,36,39-43] had more than 2 arms, but, with the exception of one study [39], only 2 arms met the inclusion criteria (see Table 1). One study was a factorial RCT where half of the participants received an engagement strategy while the other half did not [37]. One study had 7 arms assessing the effect of different timing and content of strategies [44]. The remaining 4 studies were RCTs with 2 arms [38,45-47].

All of the studies were conducted online and some studies specifically mentioned the location of the participants: the Netherlands [44,46], Australia [36,43,47], the United States [11,35,37,38,41], and Switzerland and Germany [40]. Six of the studies aimed to evaluate the effect of adding the strategy on the effectiveness of the DIs [35,39-42,47], 3 of the studies aimed to evaluate the effect of the technology-based strategies on promoting engagement with the DI [37,45,46], and 2 studies aimed to evaluate the effect of the strategy on digital intervention outcomes and engagement [36,43]. One study evaluated the effect of different timing and content of strategies on engagement [44], one study evaluated the effect of adding online peer coaching on increasing participation with a DI [38], and the final study explored the qualities of engagement with a DI [11].

**Table 1:** Characteristics of included studies.

Study	Digital intervention	Study design, engagement strategy, and comparator
Berger et al [39]	Internet-based self-help guide targeting social phobia	Three-arm RCT^a^ (75 participants included in review) Arm 1: Emails (n=24); Arm 2: Emails + telephone (n=25); Comparator: No strategy (n=26)
Berger et al [40]	Internet-based self-help program targeting depression	Three-arm RCT (50 participants included in review), one arm excluded Arm 1: Emails (n=25); Comparator: No strategy (n=25)
Clarke et al [35]	Pure self-help program targeting depression	Three-arm RCT (155 participants included in review), one arm excluded Arm 1: Telephone (n=80); Comparator: Mail (n=75)
Couper et al [11]	Tailored Web program targeting health promotion (ie, intake of fruits and vegetables)	Three-arm RCT (1677 participants included in review), one arm excluded Arm 1: Emails (n=838); Comparator: No strategy (n=839)
Farrer et al [36]	Web intervention targeting depression	Four-arm RCT (83 participants included in review), 2 arms excluded Arm 1: Telephone (n=45); Comparator: No strategy (n=38)
Greaney et al [41]	Website targeting self-monitoring of physical activity, red meat intake reduction, fruit and vegetable consumption, daily multivitamin use, and smoking cessation	Two-arm RCT and one nonrandomized arm excluded (86 participants included in review) Arm 1: Emails + telephone (n=50); Comparator: Emails (n=36)
McClure et al [37]	Internet intervention targeting smoking cessation	Randomized factorial trial (1865 participants included in review) Arm 1: Emails (n=933); Comparator: No strategy (n=932)
Muñoz et al [42]	Web-based intervention targeting smoking cessation	Four-arm RCT (498 participants included in review), 2 arms excluded Arm 1: Emails (n=251); Comparator: No strategy (n=247)
Proudfoot et al [43]	Online psychoeducation program targeting bipolar disorder	Three-arm RCT (273 participants included in review), one arm excluded Arm 1: Emails (n=134); Comparator: No strategy (n=139)
Santucci et al [45]	An entirely automated and tailored Web-based intervention targeting anxiety and depression	Two-arm RCT (43 participants included in review) Arm 1: Emails (n=21); Comparator: No strategy (n=22)
Schneider et al [46]	Computer-tailored program targeting multiple health behaviors: physical activity, fruit and vegetable intake, smoking cessation, and decreasing alcohol consumption	Two-arm RCT (3448 participants included in review) Arm 1: Emails (n=1790); Comparator: No strategy (n=1658)
Schneider et al [44]	Internet-delivered computer-tailored program targeting multiple health behaviors: physical activity, fruit and vegetable intake, smoking cessation, and decreasing alcohol consumption	Seven-arm RCT (240 participants included in review) Arms 1-3: Email at 2, 4, or 6 weeks (n=34, 34, and 35, respectively); Arms 4-6: Email with updated content at 2, 4, or 6 weeks (n=36, 35, and 32, respectively); Comparator: No strategy (n=34)
Simon et al [38]	An interactive online program targeting bipolar disorder	Two-arm RCT (118 participants included in review) Arm 1: Email (n=64); Comparator: No strategy (n=54)
Titov et al [47]	A computer-delivered treatment targeting social phobia	Two-arm RCT (163 participants included in review) Arm 1: Telephone + emails + text messages (n=81); Comparator: Emails + text messages (n=82)

^a^RCT: randomized controlled trial.

### Digital Interventions

The digital interventions targeted different health behaviors and conditions. Eight DIs were designed to target different mental health conditions, including social phobia [39,47], depression [35,36,40,45], anxiety [45], and bipolar disorder [38,43]. The rest of the DIs promoted a variety of health behaviors, including smoking cessation (n=4), decreasing alcohol consumption (n=2), self-monitoring of healthy behaviors (n=1), physical activity (n=2), and healthy diet (n=3) [11,37,41,42,44,46]. Most of the studies included detailed descriptions of the DIs. Two DIs were described as self-help guides with modules presented in a sequential order and participants could complete the whole program at once or over time [39,40]. Six DIs were composed of sessions that were presented in a sequential and phased order [11,36,43-46]. There were 2 studies that updated their DIs with new information [44,46], and 2 described their DIs as interactive [35,38].

### Technology-Based Engagement-Promoting Strategies and Their Characteristics

#### Timing

Four studies used their strategies at different time points. One engagement strategy was used at weeks 2 and 3 from baseline [41], one was used for the first 2 months postenrollment [37], one was used once on the third month from baseline and measured engagement at month 4 from baseline [46], and the last study tested the use of the strategy at multiple time points (ie, second, fourth, or sixth week from baseline) [44].

#### Duration

Strategies were used either for the duration of the DI [11,36,38-40,42,43,45,47] or at specific times [35,37,41,44,46].

#### Frequency

Most of the studies reported using engagement strategies on a regular basis. Six studies used the strategy at least once per week [36,37,39,40,45,47], one used it for 2 weeks [41], one used it three times [35], and one used it to encourage users to complete sessions with up to 4 email prompts for each session [11]. Three studies reported variable frequencies [38,42,43] and 2 studies used a strategy once only [44,46].

#### Mode of Delivery

Email was the most commonly used mode of delivery among the different studies [11,37,38,40,42-46]. Telephone calls were used in 2 studies [35,36] and 3 studies used different modes of delivery: either telephone calls in addition to emails [39,41] or telephone calls, emails, and text messages [47].

#### Sender

Other characteristics that were identified were the type of sender or provider and whether the strategies were automated [38,42] or human supported. For the latter, therapists or counsellors [11,36,39,40], nonclinical staff [35], research staff [45,47], trained coaches [41], and trained peers [43] were usually the senders or providers.

#### Content

The content of the strategies was classified into 5 types: offering assistance with the DI [35,36,39-41], advertising or describing DI content [35,44,46], linking users to specific DI pages or sections [38,42,43], reminding or inviting users to complete their DI sessions [37,44-47], and providing support and feedback on the health behavior/health problem or engagement with the DI [11,39,40,43]. Some studies described the content of their strategies in a way that enabled coding them as BCTs. The BCTs used were *social support (unspecified)* [37,39,40,43,47], *prompts/cues* where strategies explicitly prompted the users to revisit the DI [37,42,45,46], *providing feedback on behavior* (ie, engagement) [39-41], using *social reward* in the form of written encouragement and praise on participants' progress in the DI [39,40,47], *providing feedback on the outcome of behavior* (ie, engagement) in terms of the improvement in their health [39,40], and *providing instructions on how to perform the behavior* (ie, engage with a DI, such as how to log in) [35].

#### Use of Theory

No paper provided information about any underlying theoretical framework for the use, delivery, or content of strategies.

#### Tailoring

Tailoring was reported in 3 studies. In one study, participants received reports about the frequency of their usage of the DI via emails [41], and in 2 studies, participants were sent emails with personalized greetings [44,46]. Four studies described strategies that can potentially be labeled as tailored: 2 studies sent personalized feedback about progress in DI sessions to their participants [39,40], one reported using peer coaches to provide personalized advice via email to participants on how to use the materials provided through the DI [43], and one sent emails to users keyed to their smoking quit dates [42].

### Quality of Studies

The studies differed in the way they were conducted and some did not provide sufficient information to judge their quality. All studies reported randomization but only 9 reported adequate sequence generation process [35,37,39,40,42-44,46,47]. Ten studies had adequate allocation concealment [35-37,39,40,42-44,46,47]. One study reported that participants and researchers were blinded [43]. Engagement measures were prespecified in 11 studies [11,36-38,40-46], however, 3 studies out of these did not report some engagement outcomes for the intervention and control group separately [11,37,42]. Engagement measures were measured objectively, so no bias was identified for any of the studies in terms of incomplete outcome data except for one study where engagement measures were not reported for 6 participants who dropped out [39]. Protocols were only reported in 3 studies [36,37,46].

### Evaluating the Effectiveness of Technology-Based Engagement-Promoting Strategies

#### Technology-Based Engagement Strategies Compared to Minimal or Inactive Comparators

Data suitable for meta-analysis were only available for the comparison of a technology-based engagement strategy with no strategy. Two meta-analyses were performed, using dichotomous and continuous outcomes. The outcome measures of the studies included in the meta-analyses were number of DI modules/sessions/lessons completed, number of participants who completed DI modules/sessions/lessons, and number of participants who logged in/visited the DI; the outcome measures for the rest of the studies can be found in Multimedia Appendix 5.

**Figure 2:**
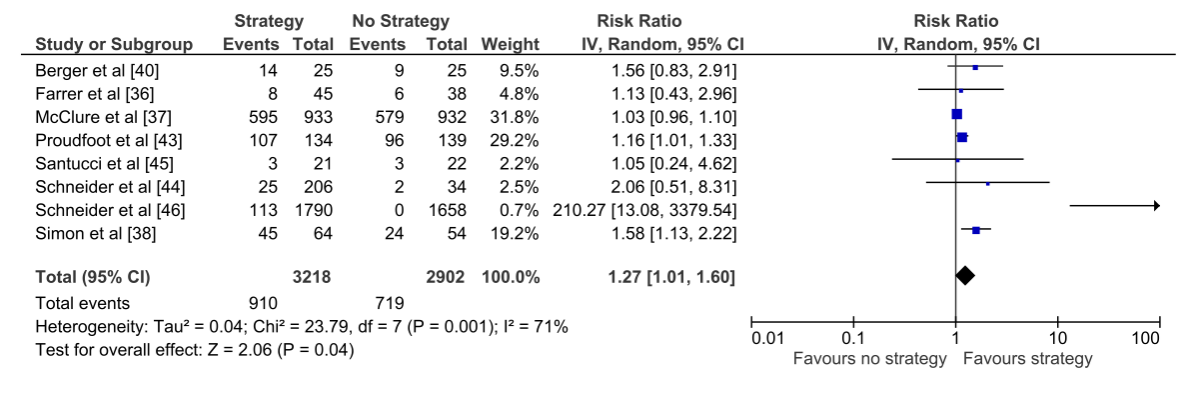
Analysis 1.1. Technology-based engagement strategy compared to no strategy: dichotomous outcomes.

Eight studies with 6120 participants reported sufficient data to be included in the meta-analyses, comparing a technology-based engagement strategy to no strategy using dichotomous outcomes (Analysis 1.1) (see Figure 2). This analysis showed that participants using DIs who received technology-based strategies were found to be significantly more likely to engage with the DI compared to those who did not receive any strategy (RR 1.27, 95% CI 1.01-1.60). However, the analysis demonstrated substantial heterogeneity between the findings of the included trials (I^2^=71%), implying that the results from the included studies differed more than would be expected by chance. Visual inspection of the forest plot suggested that the Schneider et al study [46] was an outlier. This trial had a single email prompt at 3 months, which was much later than strategies used in other studies [46]. Sensitivity analysis, excluding the Schneider et al study [46] from the forest plot, reduced the heterogeneity (I^2^=39%) and the effect of the technology-based strategy (RR 1.16, 95% CI 1.01-1.33) as shown in Multimedia Appendix 3.

**Figure 3:**
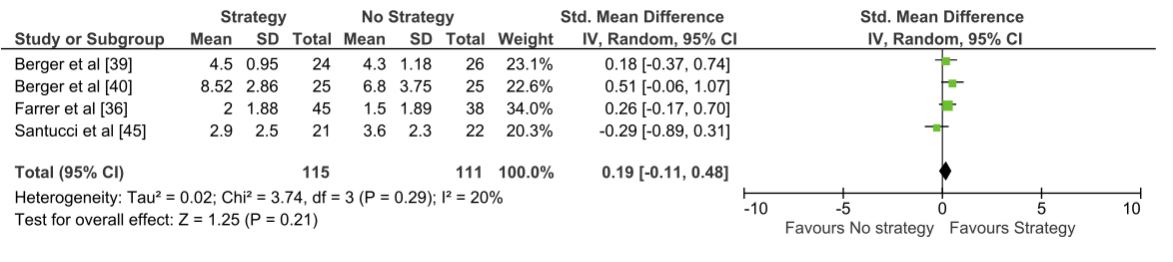
Analysis 1.2. Technology-based engagement strategy compared to no strategy: continuous outcomes.

Figure 3 shows the results of the meta-analysis for a technology-based engagement strategy compared to no strategy using continuous outcomes (Analysis 1.2). Four studies with 226 participants were included, 3 of which were included in the previous meta-analysis, and no statistically significant difference was found in engagement with a DI between participants who received technology-based strategies compared to those who did not receive any strategy (SMD 0.19, 95% CI -0.11 to 0.48). Heterogeneity was low (I^2^=20%). There is an overlap in these meta-analyses, as 3 out of the 4 studies in Analysis 1.2 were also included in Analysis 1.1; however, the direction of effect in both meta-analyses was similar.

#### Technology-Based Engagement Strategies Compared to Nontechnological Strategies and Multiple Strategies

For the other comparator types, for which a meta-analysis was not performed, one study compared technology-based engagement strategies to nontechnological means of engagement (ie, comparing telephone calls to postal mail). The postal mail group had an average of 5.9 visits and the telephone call group had an average of 5.6 visits (mean difference = 0.3 visits, *P*=.65), suggesting no statistically significant difference in outcome between the groups [35].

As for the multiple strategies group, 3 studies had 2 arms with the same technology-based engagement strategy and one of the arms received an extra strategy delivered through telephone calls. None of the studies reported a significant difference in the effect of using multiple strategies on engagement [39,41,47]. However, no conclusions can be drawn for either comparator types, as meta-analysis was not possible due to the low number of studies.

#### Characteristics of Technology-Based Engagement Strategies

No conclusions can be drawn about the effect of the different characteristics, as only one study compared the effects of timing and content of strategies on engagement with a DI. The study found that strategies sent early and those that showed DIs' updated content were more likely to engage users [44].

### Adverse and Economic Outcomes

Data on adverse and economic outcomes were intended to be extracted; however, none of the included studies reported these outcomes.

### Unpublished Data

All authors were contacted to provide and confirm information about missing or unclear engagement outcome information or characteristics of strategies, and 4 authors replied. Farrer et al provided the mean and standard deviation of BluePage visits and time spent, and more information about the strategy, including the fact that it was not tailored [36]. McClure et al provided the exact number of people allocated to the strategy and the fact that the strategy was used for 12 months [37]. Clarke et al [35] and Simon et al [38] both confirmed the accuracy in categorizing their strategies’ characteristics.

## Discussion

### Principal Findings

Technology-based strategies to promote engagement are an emerging field of research as shown by the number of included studies and their dates of publication. Generally, studies report borderline small-to-moderate positive effects of technology-based strategies on engagement compared to using no strategy, which support the use of technological strategies to improve engagement. However, this result should be treated with caution due to the high heterogeneity, small sample sizes, and the lack of statistical significance in the analysis of continuous outcomes. There were insufficient studies to effectively explore reasons for heterogeneity. No firm conclusions were drawn about which characteristics of strategies were associated with effectiveness, and due to the absence of data, no conclusions could be drawn about costs or cost-effectiveness. Although the review aimed to investigate the cost-effectiveness of engagement strategies, none of the included papers reported cost data.

To our knowledge, this is the first systematic review that evaluated technology-based engagement-promoting strategies, using website metrics as outcome measures. Other systematic reviews [21-23] investigated the effect of technological engagement strategies of DIs on behavior change and some looked at engagement-promoting features of DIs, including the use of emails and telephone calls on the change in website metrics [25]. All of these systematic reviews reported a potentially positive effect of engagement strategies on changing health behavior and engagement. However, Brouwer et al, who used similar outcome measures, did not do a meta-analysis due to the heterogeneity of the outcome measures [25].

The findings in this review agree with previous reviews that technology-based strategies may potentially promote engagement, but that there is substantial heterogeneity, potentially due to the different outcome measures used [16,25,50,51], characteristics of the DI, and engagement strategies. In this systematic review, the measures were categorized into continuous and dichotomous outcomes, and outcomes were selected for meta-analysis using prespecified criteria. This allowed for performing two meta-analyses that shared similar studies but different measures. The two meta-analyses showed a similar direction of effect.

Authors often report multiple measures of engagement, and these often vary between studies. As measures of engagement are likely to vary depending on the research question, characteristics of the engagement strategy, and the DI, clear guidance for the optimal reporting of engagement is urgently needed. Researchers need to describe and detail clearly how a DI is intended to achieve its outcomes, the level of engagement intended or desired, and the rationale for that. For example, consider a structured and session-based DI targeting a mental disorder with an email prompting users to complete all the sessions to benefit from the DI, and the research question measuring how many participants completed all the sessions—an appropriate engagement measure would be the number of participants completing all the sessions rather than number of visits or time spent on the DI.

Authors should also clearly define their concept of optimal engagement in future studies, specifying a primary outcome for engagement and the rationale for choosing it. This is supported by the fact that the other systematic reviews of engagement reported that one of the most common reasons for excluding studies is a lack of reported engagement outcomes [19,25]. Another issue related to engagement measures is the extent/duration or level of engagement that defines whether a user is successfully engaging with a DI or not. One attempt to quantify engagement was done by Kelders et al in a systematic review, which stated that a typical DI will have 50% of users engaged in it, using it at least once a week and up to 10 weeks. More research is needed to identify whether an outcome such as duration/level of engagement is enough to produce a positive effect size that justifies the cost of developing and implementing DIs [19].

This review identified themes in terms of characteristics of strategies to enable future research to selectively evaluate the different characteristics. Future primary studies that aim to determine the effectiveness of technological strategies on engagement with DIs should include a detailed description of the characteristics of engagement strategies, specifically the content of these strategies, and whether using different BCTs influence effectiveness. For this description, researchers could use the categories in this review, or expand on them. Researchers should also report the context (eg, characteristics of the DI) and outcome measures that contribute to heterogeneous results. This can help when conducting meta-analyses of future systematic reviews [52]. In addition, researchers should report multiple measures of outcome over the duration of the DI and not only report the engagement measure postintervention.

Researchers should also differentiate between attrition from the trial (ie, dropout attrition or loss to follow-up) and disengagement from the DI (ie, nonusage attrition), because studies have shown that the relation between these different types of attrition are complex and they do not share the same associated factors [18,20]. Disengagement is likely to impact on the effectiveness of the DI. It may be related to characteristics of the intervention (eg, design, usability, and perceived effectiveness) or to characteristics of the user (eg, motivation, self-efficacy, and resources). Loss to follow-up affects the ability of the study to answer the research question posed, with poor follow-up rates negatively impacting both the precision and the robustness of any estimate of effect.

### Methodological Issues

The main strengths of this review are the rigorous and systematic methodology, which followed Cochrane methodological guidance, and the comprehensive and extensive search strategy. Furthermore, screening, extraction, and risk of bias assessment were independently conducted or reviewed by at least two authors. The review also includes meta-analyses to measure the effect of using the strategies compared to no strategies. In addition, the published, peer-reviewed protocol provides transparency.

The systematic review included RCTs as the most rigorous method for evaluating strategies, however, it is increasingly being recognized that the inclusion of other types of research is important. Policy makers and researchers are facing complex questions that the rigid and quantitative types of studies might not answer most appropriately. Rather, qualitative studies might be more equipped to fill in the gaps that RCTs cannot provide an answer for, such as the experiences of participants, the possible contradiction in some outcomes, and theory development [53]. In the case of engagement, certain issues can only be answered through conducting qualitative studies rather than quantitative ones [54]. These issues may include understanding what outcomes mean for the user (eg, DI visits, page views, and time spent on the DI), what the experience of the engaged user is compared to the disengaged user, and the preference of users.

The limited search of the grey literature might be considered a limitation; however, in the case of this emerging field of research, the risk of significant publication bias is probably low because both negative and positive findings are of interest. A funnel plot could have been used to estimate the degree of publication bias; however, this was not possible because of the low number of studies, and the possibility of funnel plot asymmetry due to the different methodological qualities of the studies regardless of the existence of publication bias [33]. Another possible limitation might be that the use of the current Cochrane bias assessment guidelines might be more suitable for generic drug trials as opposed to DIs. For example, sequence generation is not an issue as judged in this review, as it is made easier with the use of online randomization programs. Blinding of staff and participants might not be possible as the control and intervention groups may be aware of receiving strategies sent by the staff. Criteria for traditional outcome assessment might not be suitable for reviewing studies of engagement, as it has to be tailored to how engagement is measured (eg, by automatic website metrics). For most of the studies, the description provided was not sufficient to judge the different aspects of trial quality. Authors and developers of DIs can benefit from using the enhanced CONSORT-EHEALTH reporting guide, published by JMIR. It can help clarify what authors need to report and describe in their studies to enable readers and reviewers to judge a study’s quality [55].

### Conclusions

Technology-based strategies may promote engagement compared to using no strategy; however, this finding should be interpreted with caution as only a small number of eligible studies were identified for the meta-analysis and the results were heterogeneous. The field of engagement strategies is an emerging field, as indicated by the number and dates of the studies; more research is needed to understand what strategy characteristics are effective and how cost-effective they are.

## Appendix

Multimedia Appendix 1 MEDLINE search strategy.

Multimedia Appendix 2 (A) Risk of bias summary. (B) Risk of bias graph.

Multimedia Appendix 3 Comparison engagement strategy versus no engagement strategy: dichotomous outcomes sensitivity analysis after removing the study by Schneider et al [46].

Multimedia Appendix 4 Detailed characteristics of included studies.

Multimedia Appendix 5 Main engagement outcomes and findings reported in included studies.

## Abbreviations

BCT: behavior change technique
CENTRAL: Cochrane Central Register of Controlled Trials
CINAHL: Cumulative Index to Nursing and Allied Health Literature
DI: digital intervention
ERIC: Education Resources Information Center
JMIR: Journal of Medical Internet Research
PRISMA: Preferred Reporting Items for Systematic Reviews and Meta-Analyses
RCT: randomized controlled trial
RR: relative risk
SMD: standardized mean difference
UCL: University College London
