# Supplementary material for: The Effectiveness of Prompts to Promote Engagement With Digital Interventions: A Systematic Review
Source: J Med Internet Res. 2016 Jan 8;18(1):e6. doi: 10.2196/jmir.4790 (PMC4723726; doi:10.2196/jmir.4790)
Supplement: Multimedia Appendix 2 [file jmir_v18i1e6_app2.pdf]

	Random sequence generation (selection bias)	Allocation concealment (selection bias)	Blinding of participants and personnel (performance bias)	Incomplete outcome data (attrition bias)	Selective reporting (reporting bias)	Other bias
Berger et al [39]	+	+	−	?	?	+
Berger et al [40]	+	+	?	+	+	+
Clarke et al [35]	+	+	−	+	?	+
Couper et al [11]	?	?	?	+	?	+
Farrer et al [36]	?	+	?	+	?	+
Greaney et al [41]	?	?	−	+	+	+
McClure et al [37]	+	+	?	+	?	+
Munoz et al [42]	+	+	−	+	?	+
Proudfoot et al [43]	+	+	+	+	+	+
Santucci et al [45]	?	?	?	+	+	+
Schneider et al [44]	+	+	?	+	+	+
Schneider et al [46]	+	+	−	+	?	+
Simon et al [38]	?	?	?	+	+	+
Titov et al [47]	+	+	?	+	?	+
