# Supplementary figures and images for: The Effectiveness of Prompts to Promote Engagement With Digital Interventions: A Systematic Review
Source: J Med Internet Res. 2016 Jan 8;18(1):e6. doi: 10.2196/jmir.4790 (PMC4723726; doi:10.2196/jmir.4790)

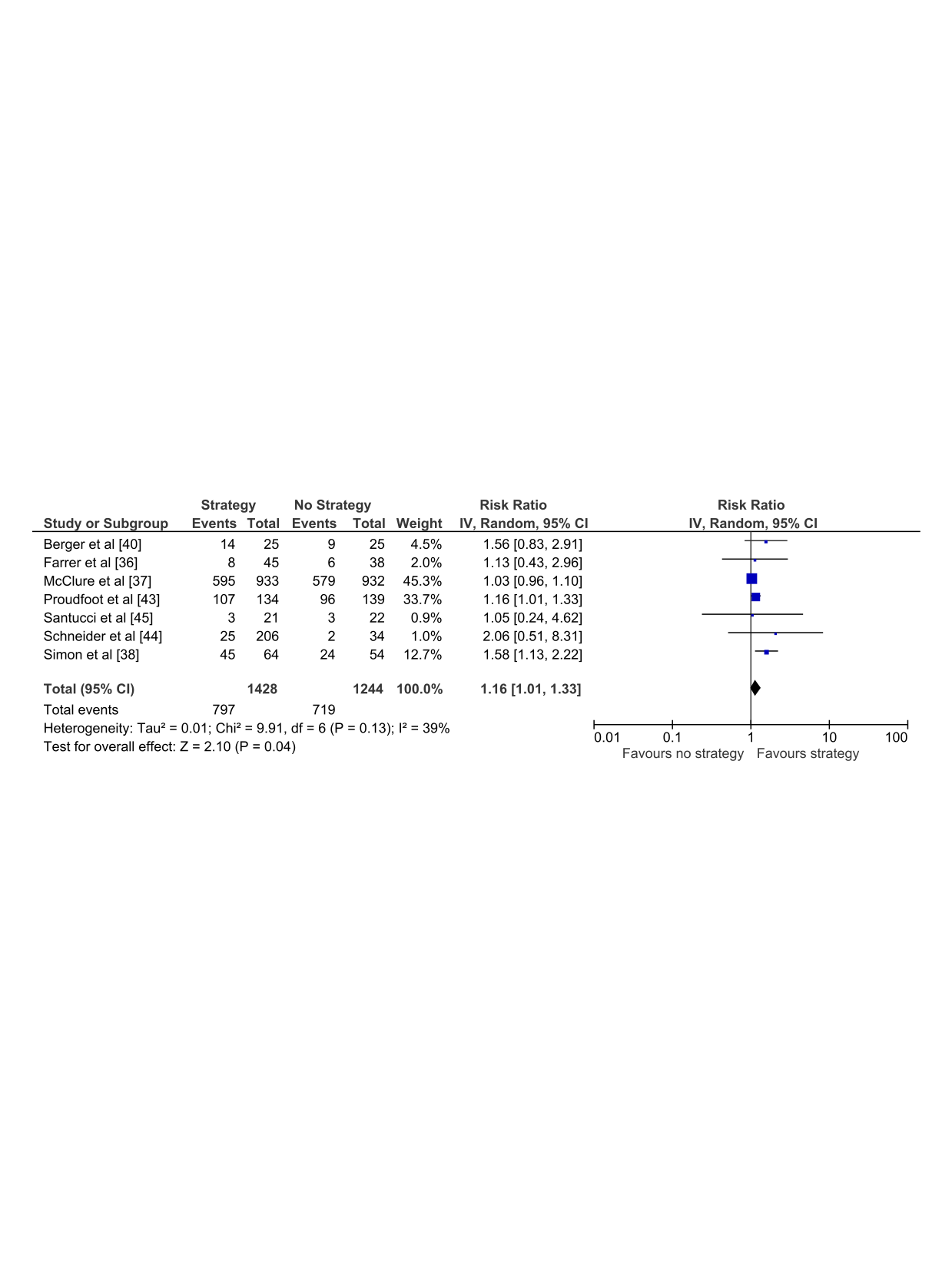

Supplement: Multimedia Appendix 3 [file jmir_v18i1e6_app3.png]
