# Supplementary material for: The Effectiveness of Prompts to Promote Engagement With Digital Interventions: A Systematic Review
Source: J Med Internet Res. 2016 Jan 8;18(1):e6. doi: 10.2196/jmir.4790 (PMC4723726; doi:10.2196/jmir.4790)
Supplement: Multimedia Appendix 4 [file jmir_v18i1e6_app4.pdf]

Study	Participants, study designs and arms	Digital intervention	Engagement strategy and comparator
Berger et al [39]	**Participants:** 81 were randomized, six dropped out, 75 were included in the review.<br>**Study design:** RCT (three arms).<br>**Included arms:** pure self-help (n=26), guided self-help (n=24) and step-up of support on demand (n=25).	**Name:** Not mentioned<br>**Target health behavior/ health condition:** social phobia<br>Internet-based self-help guide. Comprised five largely text-based lessons, several exercises and diaries and the option to participate in an online discussion forum.<br>Participants had to work through the program in a sequential order but all lessons could be completed at once.	**Engagement strategy**<br>**Arm 1: guided self-help**<br>**Mode of delivery**: email<br>**Content**: the therapists introduced themselves through email and informed participants that they could contact them whenever they wanted to.<br>Therapists also sent an email with feedback to the participants on their behavior and progress in the self- help guide. In case there was no activity by a participant during the week, therapists offered their help and assistance and asked if he or she was facing any problem with the program or with the tasks.<br>**Frequency, duration and timing:** weekly contact for the duration of the DI (ten weeks).<br>**Sender:** psychology therapist<br>**Arm 2: step-up of support on demand Mode of delivery**: email+ telephone<br>**Content:** at the end of each session of the DI, participants were automatically asked if they would like to receive additional support from a therapist. In a first step, and simply by clicking on a button, participants could ask for contact with a therapist via email. In a second step, and after the email contact had been established, participants were asked at the end of each session if they would like to have additional

			weekly telephone calls with their therapist. In addition, automated standardized emails were sent to participants in case they did not use the program for more than seven days. These emails informed recipients about the possibility of an additional contact with a therapist via email (in a first step) or via telephone (in a second step). Participants were able to click on a link in the email if they wanted this additional contact.<br>**Frequency, duration and timing:** weekly contact (by choice) for the duration of the DI (ten weeks).<br>**Sender:** psychology Therapist<br>**Comparator arm: pure self-help**<br>This group did not receive any engagement strategy.
Berger et al [40]	**Participants:** 76 were randomized, 50 were included in review.<br>**Study design:** RCT (three arms).<br>**Included arms:** unguided self- help (n=25), guided self-help(n=25).<br>**Excluded arm:** wait-list control (n=26).	**Name:** Deprexis<br>**Target health behavior/health condition:** depression<br>Internet- based self- help program. Consisted of ten content modules and a summary module covering a variety of therapeutic content. Participants were encouraged to work through the program in a sequential order. Modules were not made available sequentially, thus participants could complete the whole program at once.	**Engagement strategy arm: guided self-help**<br>**Mode of delivery**: email<br>**Content:** the therapists introduce themselves and informed participants that they could contact them whenever they wanted to. Once a week, therapists wrote a short e-mail with feedback based on participants' program usage over the previous week. The feedback did not refer to specific text passages or responses participants had selected. It was relatively generic in nature and did not discuss specific therapeutic strategies in detail. In case there was no activity by a participant, therapists offered their help

			and assistance and asked if the participant was facing any problem with the program or with the tasks.<br>**Frequency, duration and timing:** weekly contact for the duration of the DI (ten weeks).<br>**Sender:** psychology therapist<br>**Comparator arm: unguided self-help**<br>This group did not receive any engagement strategy.
Clarke et al [35]	**Participants:** 255 were randomized, 155 were included in the review.<br>**Study design:** RCT (three arms).<br>**Included arms:** mail reminders (n=75), telephone reminders (n=80).<br>**Excluded arm:** treatment as usual (n=100).	**Name:** Overcoming Depression on the Internet (ODIN)<br>**Target health behavior/health condition:** depression<br>A pure self-help program offering training in cognitive restructuring. The intervention was organized in seven "chapters," each presenting a new technique via interactive examples and practice opportunities.	**Engagement strategy arm: telephone reminders**<br>**Mode of delivery**: telephone<br>**Content:** brief (< 5 minutes) telephone reminders from non-clinician study staff where they first identified themselves and the study, then reminded participants of the ODIN website address and gave instructions for looking up forgotten passwords. They read a brief description of a feature of the website designed to entice the participant to make a return visit then concluded the call.<br>**Frequency, duration and timing:** Staff called at two, eight, and 13 weeks after enrolment.<br>**Sender:** The reminder staff had no mental health background, and they were prohibited from engaging in any therapy-like activity. Staff were capable of, and limited to, answering questions only about basic website troubleshooting (e.g. difficulty logging on).<br>**Comparator arm: mail postcard reminders**

			**Delivery channel:** mail postcards<br>**Content:** The content was scripted to convey information identical to that included in the telephone reminder calls.<br>**Frequency, duration and timing:** Mail was sent at two, eight, and 13 weeks after enrolment.<br>**Sender:** Reminder staff who had no mental health background.
Couper et al [11]	**Participants:** 2513 were randomized, 1677 were included in the review.<br>**Study design:** RCT (three arms).<br>**Included arms:** tailored DI (n=839), tailored DI +human online behavioral intervention (HOBI) (n=838).<br>**Excluded arm:** untailored DI (n=836).	**Name:** Making effective nutritional choices for cancer prevention (MENU)<br>**Target health behavior/health condition:** health promotion (intake of fruits and vegetables). The DI offered four core tailored education sessions phased over a four months period.	**Engagement strategy arm: tailored DI+HOBI**<br>**Mode of delivery:** email<br>**Content:** email counselling support sessions.<br>Each counselling session was initiated by a study counsellor within a week after each web session was first visited. Counsellors provided additional support for dietary change and responded to any request for strategies or for nutrition information with a referral to the MENU website.<br>**Frequency, duration and timing:** a maximum of four unique email discussions corresponding to each of the four web sessions were initiated by the counsellor when the sessions were accessed. Each email discussion was limited to four “back and forth” exchanges and the duration of these emails was based on the web sessions which was a four month period.<br>**Sender:** Counsellors<br>**Comparator arm: tailored DI**<br>This group did not receive any engagement strategy.

Farrer et al [36]	**Participants:** 155 were randomized, 83 were included in the review.<br>**Study design:** RCT (four arms).<br>**Included arms:** web only (n=38), web with telephone tracking (n=45)<br><br>**Excluded arms:** telephone tracking only (n=37), control (n=35).	**Name:** BluePages andMoodGYM<br>**Target health behavior/health condition:** depression<br>The web only intervention delivered online psychoeducation on the first week provided by BluePages (bluepages.anu.edu.au) combined with cognitive behavioral therapy on the second to sixth weeks provided by MoodGYM (moodgym.anu.edu.au.)<br>BluePages is a freely accessible, psychoeducational website that contains information and resources related to depression.<br>The MoodGYM program is a free to end-user, online program for depression. The program is divided into five modules designed to be completed sequentially.	**Engagement strategy arm: web with phone tracking**<br>**Mode of Delivery:** telephone call<br>**Content:** telephone call addressing any issues associated with the participants' use of the online programs.<br>**Frequency, duration and timing:** weekly ten minutes calls for six weeks.<br>**Sender:** telephone counsellor<br>**Comparator arm: web only**<br>This group did not receive any engagement strategy.
Greaney et al [41]	**Participants:** 86 were randomized and included in the review.<br>**Study design:** RCT (two arms and one non- randomized arm).<br>**Included arms:** automated assistance (AA) (n=36), automated assistance + calls (AAC) (n=50).<br><br>**Excluded arm:** observation only (OO) (n=14).	**Name:** Healthy Directions 2<br>**Target health behavior/health condition:** targets self- monitoring of the following health behaviors (1) promote physical activity, (2) reduce red meat intake, (3) increase fruit and vegetable consumption, (4) promote daily multivitamin use, and (5) promote smoking cessation<br>The website included a user-friendly section where patients could self-monitor all targeted behaviors at once. The website allowed participants to enter data for the day they logged into the website and for the two days prior. After entering data, participants received immediate feedback in the form of graphs and descriptive text. Participants could also view their data over time, to assess overall progress.	**Engagement strategy arm: AAC**<br>**Mode of delivery:** emails +telephone calls<br>**Content:** participants received emails that encouraged them to track their behaviors via the study website. Email messages changed daily and included a brief message about the benefits of self- monitoring and a hyperlink to the study website. Participants also received two tailored self-monitoring reports that provided feedback to the individual about his or her frequency of tracking for each of the behaviors during the previous week. Reports were viewed as part of the prompting intervention. If participants did not self-monitor, their reports reiterated the information on the benefits of self-monitoring that was included on the daily emails and encouraged self- monitoring via the study website. Participants also received two technical assistance calls. The calls were designed to be brief (< 5 minutes) and focused on troubleshooting technical questions (e.g.

			trouble logging in or how to self- monitor on the website).<br>**Frequency, duration and timing:** participants received two weeks of daily emails during the prompting period (weeks two and three) and two tailored self- monitoring reports: the first at the end of week two and the second at the end of week three The telephone calls; the first call was made at the end of the first week of prompting (week two) and the second call took place at the end of the second week of prompting (week three).<br>**Sender:** calls were made by a trained health coach.<br>**Comparator arm: AA**<br>**Mode of delivery:** emails<br>**Content:** Emails were similar to those received by participants in the AAC condition.<br>**Frequency, duration and timing:** participants received two weeks of daily emails during the prompting period (weeks two and three) and two tailored self- monitoring reports: the first at the end of week two and the second at the end of week three.
McClure et al [37]	**Participants:** 1865 were randomized and included in the review<br>**Study design:** randomized Factorial Trial assessing four design intervention features (including use of email reminders).<br>**Included arms:** no proactive email reminders (n=932), proactive email reminders (n=933).	**Name:** Q2<br>**Target health behavior/ health condition:** smoking cessation<br>The intervention was tailored to user's needs and delivered via the Internet. The intervention included a combination of core intervention content and additional special feature content. The core content was accessible from the main page and organized in three main drop-down headers or content areas, each targeting smokers at different stages of readiness to quit smoking.	**Engagement strategy arm: proactive email reminders**<br>**Mode of delivery:** emails<br>**Content:** email messages were standardized across all individuals and encouraged participants to return to the Q2 website to view the optional special feature content.<br>**Frequency, duration and timing:** weekly (for two months).<br>**Comparator arm: no proactive email reminders**<br>This group did not receive any engagement strategy.
Munoz et al [42]	**Participants:** 1000 were randomized , 498 were included in the review.	**Name:** Guía para Dejar de Fumar (Guide to Stop Smoking,"Guía")<br>**Target health behavior/health condition:** smoking cessation	**Engagement strategy arm: Guía+ ITEMs**<br>**Mode of delivery:** emails

	**Study design:** Parallel-groups RCT (four arms).<br>**Included arms:** Guía (n=247), Guía+ Individually Timed Educational Messages (ITEMs) (n=251)<br>**Excluded arms:** Guía +ITEMs +mood management course (n=251) and Guía+ ITEMs+ mood management course+ virtual group (n=251).	Web-based intervention "Guía", a National Cancer Institute evidence-based intervention initially developed for Spanish-speaking smokers. It contained an online static "Guía," a cigarette counter, and an online journal to record experiences while quitting.	**Content:** automated emails with links to the Guía keyed to quit date.<br>**Frequency, duration and timing:** timed to quit date for approximately eight weeks.<br>**Sender:** automated.<br>**Comparator arm: Guía**<br>This group did not receive any engagement strategy.
Proudfoot et al [43]	**Participants:** 419 were randomized , 273 were included in review.<br>**Study design:** RCT (three arms).<br>**Included arms:** Bipolar Education Program (BEP) (n=139), BEP+Informed Supporters (IS) (n=134).<br>**Excluded arm:** control (n=134).	**Name:** BEP<br>**Target health behavior/ health condition:** bipolar disorder<br>The online psychoeducation program was an eight session audio–visual program. The program topics were standard for all users and presented in a sequential, non-interactive manner. Behavioral tasks for completion between online sessions were added in this study to facilitate application and practice of the content. One module per week was sent to participants over eight weeks.	**Engagement strategy arm: BEP+IS**<br>**Mode of delivery:** emails<br>**Content:** email coaching by people with bipolar disorder. Emails were designed to answer any questions participants may have had and to provide examples of how to apply the education material in the website to their everyday lives. Emails focused on effective self-management across three domains: medical management, emotional management and role management. Emails were linked to the content of the online psycho- education program.<br>**Frequency, duration and timing:** emails to a participant from an Informed Supporter were restricted to two 300-word communications per week for eight weeks.<br>**Sender:** Informed Supporters, people with bipolar disorder who had been effectively managing their condition for at least two years.<br>**Comparator arm: BEP**<br>This group did not receive any engagement strategy.
Santucci et al [45]	**Participants:** 44 were randomized, 43	**Name:** Beating the Blues (BtB)<br>**Target health behavior/health condition:** anxiety and depression	**Engagement strategy arm: reminder**<br>**Mode of delivery:** emails

	were included in the review.<br>**Study design:** Pilot RCT (two arms).<br>**Included arms:** no reminder (n=22) and reminder (n=21).	An entirely automated and tailored web- based cognitive- behavioral intervention that consisted of eight sequential weekly sessions.The program is designed for sessions to build on one another, often providing individualization to the patient's unique needs.	**Content:** emails were sent to remind users to complete their BtB session for the week.<br>**Frequency, duration and timing:** weekly for eight weeks.<br>**Sender:** study staff.<br>**Comparator arm: no reminder**<br>This group did not receive any engagement strategy.
Schneider et al [46]	**Participants:** 3448 were randomized and included in the review.<br>**Study design:** RCT (two arms).<br>**Included arms:** no prompting (n=1658), prompting (n=1790).	**Name:** Not mentioned<br>**Target health behavior/health condition:** multiple health behaviors: physical activity, fruit and vegetable intake, smoking cessation, and alcohol consumption.<br>The Computer Tailored (CT) program used a dual approach to guide people toward behavior change. The first part consisted of a health risk appraisal and was aimed at increasing participants' awareness of their health behavior status and second part provided assistance in changing participants' health behavior by offering five separate CT modules. The modules used a fixed, gradual approach consisting of four steps, guiding people toward behavior change. The website was regularly updated.	**Engagement strategy arm: prompting**<br>**Mode of delivery:** email<br>**Content:** an email was sent to prompt users to revisit the program. This email opened with a personalized greeting and reminded people about their first visit to the program. Subsequently, people were invited to revisit the program to obtain information on their health status and to monitor their progress. Participants were also informed of the opportunity to receive additional, iterative health advice on the health behaviors selected at baseline or on a new behavior. The email also contained details on personal log-in information (user name and password). The email concluded with greetings from the research team and contact information.<br>**Frequency, duration and timing:** email was sent three months from the baseline visit.<br>**Comparator arm: no prompting**<br>This group did not receive any engagement strategy.
Schneider et al [44]	**Participants:** 240 were randomized and included in review.<br>**Study design:** RCT (seven arms).	**Name:** Internet- Delivered Computer- Tailored Lifestyle Program<br>**Target health behavior/health condition**: five health behaviors: Physical activity, fruit and vegetables intake, smoking	**Engagement strategies:**<br>**Arms 1,2 and 3: standard prompts**<br>**Mode of delivery:** email<br>**Content:** participants received an email that contained a reminder about

	**Included arms:** control condition- no prompt (NP)(n=34), six experimental conditions: three groups sent prompt with standard content (SP) at two weeks (n=34), at four weeks (n=34) , at six weeks (n=35) and three groups sent prompt with standard prompt and additional content (SP+) at two weeks (n=36), at four weeks (n=35) and at six weeks (n=32).	cessation and decreasing alcohol consumption.<br>Website with computer tailored (CT) lifestyle program embedded in it. The program used a dual approach to guide people toward behavior change. First, awareness of participants' current health behavior status was increased by comparing their status to the Dutch public health guidelines set for these health behaviors. Second, assistance was provided in changing participants' health behavior by using CT modules available per behavior. The modules used a fixed, gradual approach consisting of four steps, guiding people toward behavior change. The website was regularly updated.	user previous visit to the CT program and an invitation to visit it again to monitor their behavior change progress and obtain additional feedback and advice. This standard email opened with a personalized greeting and reminded people about their first visit to the program. Participants were also given the opportunity to receive additional iterative health advice on the health behavior(s) selected at baseline or on a new behaviour. To facilitate logging in to the program, the email also contained details about their personal log-in information (username and password). The email concluded with greetings from the research team and contact information.<br>**Frequency, duration and timing:** one email was sent to each group either on the second or fourth or the sixth week from baseline.<br>**Arms 4,5 and 6: standard prompt and additional content (SP+)**<br>**Mode of delivery:** email<br>**Content:** participants received the same email content as SP with additional content.<br>Additional content consisted of new information that was placed on the program website since they last visited it. This information referred to nutrition and provided examples of healthy food alternatives that were available for that current season (eg, spring/summer).<br>**Frequency, duration and timing:** one email was sent to each group either on the second or fourth or the sixth week from baseline<br>**Comparator arm: no prompt**<br>This group did not receive any engagement strategy.

Simon et al [38]	**Participants:** 118 were randomized and included in the review.<br>**Study design:** Pilot RCT (two arms).<br>**Included arms:** program only (n=54), coaching (n=64).	**Name:** My Recovery Plan<br>**Target health behavior /health condition:** bipolar disorder<br>An interactive online program to support people living with bipolar disorder in creating and using personal recovery plans. Participants received online coaching support from certified peer specialists, however, communication with those coaches was done within the website to protect participants' privacy.	**Engagement strategy arm: coaching**<br>**Mode of delivery:** email<br>**Content:** emails were sent to the participants alerting them every time they received a new message from their coaches. Emails did not contain sensitive information.<br>**Frequency, duration and timing:** email notifications were sent when coaches sent private messages on the website so frequency varies for different users, however those notifications were related to coaches messaging users which was for one year (the duration of the DI).<br>**Sender:** automated<br>**Comparator arm:program only**<br>This group did not receive any engagement strategy.
Titov et al [47]	**Participants:** 168 were randomized, 163 were included in review<br>**Study design:** Pragmatic RCT (two arms).<br>**Included arms:** computerized cognitive behavior therapy (CCBT) (n=82) and CCBT+ Telephone (n=81).	**Name:** Not mentioned but study name was Shyness 4<br>**Target health behavior /health condition**: social phobia<br>A computer delivered treatment that consisted of six online lessons, a summary/homework assignment for each lesson, comments by prior participants about each lesson. All participants were asked to complete the six lessons within eight weeks of starting.	**Engagement strategy arm: CCBT+Telephone**<br>**Mode of delivery:**telephone +emails+text messages<br>**Content:** participants were telephoned each week by a research assistant, at a time specified by the participant, when they were commended and encouraged to persevere but no clinical advice was offered. Each participant received four text messages and an average of 15 automated emails which were sent at specific stages of the programme (the criteria for sending an email included a participant completing a lesson, not completing a lesson within a specified time frame, or to alert participants to additional material that had been made available in relation to a specific lesson or issue).<br>**Frequency, duration and timing:** weekly telephone calls, fortnightly text and variable emails for eight weeks.<br>**Sender:** Research assistant made the calls

			**Comparator arm: CCBT**<br>**Mode of delivery:** emails +text messages<br>**Content:** each participant received four text messages and an average of 15 automated emails which were sent at specific stages of the programme (the criteria for sending an email was similar to the intervention arm).<br>**Frequency, duration and timing:** fortnightly text and variable emails for eight weeks.
