# Supplementary material for: The Effectiveness of Prompts to Promote Engagement With Digital Interventions: A Systematic Review
Source: J Med Internet Res. 2016 Jan 8;18(1):e6. doi: 10.2196/jmir.4790 (PMC4723726; doi:10.2196/jmir.4790)
Supplement: Multimedia Appendix 5 [file jmir_v18i1e6_app5.pdf]

Study (author name, year)	Included study arms	Dichotomous outcomes		Continuous outcomes	
		Outcome	Result	Outcome	Result
Berger et al [39]	pure self-help: (n = 26)<br>guided self-help : (n =24)<br>step-up of support on demand: (n=25)			Mean number of lessons completed (five lessons)	pure self-help: mean 4.3 lessons (SD = 1.18)<br>guided self-help: mean 4.5 lessons (SD =.95)<br>step-up of support on demand: mean 4.6 lessons (SD =.98)
Berger et al [40]	unguided self-help: (n = 25)<br>guided self-help: (n = 25)	Number of participants who completed the ten lessons	unguided self-help: n(%) =9 (36%)<br>guided self-help: n(%)= 14 (56%)	Mean number of lessons completed (ten lessons)	unguided self-help: mean 6.8(SD=3.75)<br>guided self-help: mean 8.52(SD=2.86)
Clarke et al [35]	mail reminders: (n=75)<br>telephone reminders: (n=80)			Mean number of log-ins	mail reminders: mean= 5.9(SD =6.2)<br>telephone reminders: mean= 5.6 (SD=5.8)
Couper et al [11]	tailored: (n = 839)<br>tailored + human online behavioral intervention (HOBI): (n = 838)			Mean total minutes devoted to the intervention website	tailored: mean = 44.1<br>tailored + HOBI : mean= 46.7
Farrer et al [36]	web only: (n=38)<br>web with tracking: (n=45)	Number of participants who completed all five moodgym modules	web only: n(%) =6 (15.8%)<br>web with tracking: n(%)=8 (17.8%)	Mean number of completed MoodGYM program modules (five modules)	web only: mean = 1.5 (SD=1.89)<br>web with tracking: mean= 2.0 (SD=1.88)
Greaney et al [41]	automated assistance (AA): (n=36)<br>automated assistance + calls (AAC): (n=50)	Number of participants who reached the minimum threshold of self-monitoring	During week two: AA: (%)=10<br>AAC:(%)=22<br>During week three: AA:(%)=14<br>AAC:(%)=26		

		at least one behavior three or more times per week during the prompting periods (week two and three)			
McClure et al [37]	no proactive email reminders : (n=932)<br>proactive email reminders : (n=933)	Proportion of participants viewing content who logged in/visited the DI	no proactive email reminders :n(%)=579 (62.12%)<br>proactive email reminders: n(%)=595 (63.88%)		
Munoz et al [42]	Guía: (n=247)<br>Guía+ Individually Timed Educational Messages (ITEMs): (n= 251)	Utilization of a cigarette counter and online journal [1]			
Proudfoot et al[43]	Bipolar Education Program (BEP): (n = 139)<br>BEP+Informed Supporters(IS): (n = 134)	Number of participants who completed and returned four or more sessions of the eight sessions program	BEP: n(%)= 96 (69.1%)<br>BEP+ IS: n(%)= 107(79.9%)		
Santucci et al [45]	no reminder: (n=22)<br>reminder: (n=21)	Number of participants who completed all eight sessions	no reminder: n=3<br>reminder: n=3	Mean number of sessions completed (eight sessions)	no reminder: mean = 3.6 (SD =2.3)<br>reminder: mean = 2.9 (SD = 2.5)
Schneider et al [46]	no prompting: (n=1658)<br>prompting: (n=1790)	Number of participants who visited the program during the one month follow up	No prompting: n(%)=0(0)<br>prompting:n(%)=113(6.3%)		

Schneider et al [44]	no prompt (NP): (n=34)<br>standard prompt (SP)at two weeks: (n=34)<br>SP at four weeks : (n=34)<br>SP at six weeks: (n=35) standard prompt and additional content (SP+) at two weeks: (n=36)<br>SP+ at four weeks: (n=35)<br>SP+ at six weeks: (n=32)	Number of participants who logged in/visited the DI	NP: n(%) =2(5.9)<br>SP at two weeks :n(%) =6(17.6)<br>SP at four weeks : n(%) =1(2.9)<br>SP at six weeks: n(%) =1(2.9)<br>SP+ at two weeks: n(%) =10(27.8)<br>SP+ at four weeks: n(%) =7(20)<br>SP+ at six weeks: n(%) =0(0)		
Simon et al [38]	program only (n=54)<br>coaching (n=64)	Number of participants who returned after initial sign up at anytime	Program only: n(%)= 24(44%)<br>coaching: n(%)=45(71%)		
Titov et al [47]	computerized cognitive behaviour therapy (CCBT): (n=82)<br>CCBT+Telephone: (n=81)			Mean number of logins	CCBT: mean= 23<br>CCBT+Telephone: Mean =23

[1]The results were not reported separately for each arm
